# Tracking COVID-19 Discourse on Twitter in North America: Infodemiology Study Using Topic Modeling and Aspect-Based Sentiment Analysis

**DOI:** 10.2196/25431

**Published:** 2021-02-10

**Authors:** Hyeju Jang, Emily Rempel, David Roth, Giuseppe Carenini, Naveed Zafar Janjua

**Affiliations:** 1 Department of Computer Science University of British Columbia Vancouver, BC Canada; 2 British Columbia Centre for Disease Control Vancouver, BC Canada; 3 School of Population and Public Health University of British Columbia Vancouver, BC Canada; 4 Centre for Health Evaluation and Outcome Sciences University of British Columbia Vancouver, BC Canada

**Keywords:** COVID-19, Twitter, topic modeling, aspect-based sentiment analysis, racism, anti-Asians, Canada, North America, sentiment analysis, social media, discourse, reaction, public health

## Abstract

**Background:**

Social media is a rich source where we can learn about people’s reactions to social issues. As COVID-19 has impacted people’s lives, it is essential to capture how people react to public health interventions and understand their concerns.

**Objective:**

We aim to investigate people’s reactions and concerns about COVID-19 in North America, especially in Canada.

**Methods:**

We analyzed COVID-19–related tweets using topic modeling and aspect-based sentiment analysis (ABSA), and interpreted the results with public health experts. To generate insights on the effectiveness of specific public health interventions for COVID-19, we compared timelines of topics discussed with the timing of implementation of interventions, synergistically including information on people’s sentiment about COVID-19–related aspects in our analysis. In addition, to further investigate anti-Asian racism, we compared timelines of sentiments for Asians and Canadians.

**Results:**

Topic modeling identified 20 topics, and public health experts provided interpretations of the topics based on top-ranked words and representative tweets for each topic. The interpretation and timeline analysis showed that the discovered topics and their trend are highly related to public health promotions and interventions such as physical distancing, border restrictions, handwashing, staying home, and face coverings. After training the data using ABSA with human-in-the-loop, we obtained 545 aspect terms (eg, “vaccines,” “economy,” and “masks”) and 60 opinion terms such as “infectious” (negative) and “professional” (positive), which were used for inference of sentiments of 20 key aspects selected by public health experts. The results showed negative sentiments related to the overall outbreak, misinformation and Asians, and positive sentiments related to physical distancing.

**Conclusions:**

Analyses using natural language processing techniques with domain expert involvement can produce useful information for public health. This study is the first to analyze COVID-19–related tweets in Canada in comparison with tweets in the United States by using topic modeling and human-in-the-loop domain-specific ABSA. This kind of information could help public health agencies to understand public concerns as well as what public health messages are resonating in our populations who use Twitter, which can be helpful for public health agencies when designing a policy for new interventions.

## Introduction

Worldwide, more than 31 million people have been diagnosed with COVID-19, and more than 1 million people have died as of October 12, 2020 [1]. Waiting for the development and rollout of a vaccine, governments across the world have implemented wide-ranging nonpharmaceutical interventions such as hand hygiene, face masks, contact tracing, isolation and quarantine, and physical (social) distancing through banning mass gatherings and lockdowns to reduce the transmission of SARS-CoV-2. The impact of COVID-19 and measures to prevent transmission has generated a lot of discussion among the general population, medical and public health professionals, and government officials [2,3]. Some of this discourse is happening on social media such as Twitter.

During this pandemic, people have been using social media such as Twitter to share news, information, opinions, and emotions about COVID-19 [4,5], similar to previous infectious disease outbreaks such as Ebola. In the Eloba outbreak, public health organizations helped contain Ebola by monitoring conversations on social media and spreading accurate information about the disease [6-9]. As we can see from these past successes, social media is an important source to learn about people’s reactions and concerns. This information can assist public health authorities in the monitoring and surveillance of health information, concerns, and behaviors, and designing interventions to reduce the impact of the pandemic. Understanding people’s information needs, misinformation, hate speech and discrimination, compliance with preventative measures, and other reactions to COVID-19, and where their concerns lie helps to tailor public health strategy and ultimately create better informed interventions.

Topic modeling and sentiment analysis have been widely used to identify issues and people’s opinions in public health and is being used to understand COVID-19–related issues as well (Table 1). Analyses were conducted to identify patterns of health communications in diverse kinds of data sources, communities, and locations. Although some works investigated news articles [10] or research papers [11], most research focused on social media such as Reddit posts [12] and tweets [13-19]. Conversations in particular communities were examined, such as tweets posted by US governors and presidential cabinet members [13], and African American twitter communities [16]. Specific languages and locations were discussed as well (eg, Chinese news articles [10], Persian and Farsi tweets in Iran [14], and English tweets in California and New York in the United States [17]). Although all these works investigated people’s reactions toward COVID-19, there have been few studies about general public responses in Canada. Furthermore, although sentiment analysis has been broadly applied [15-18], the techniques used in prior work determine the sentiment of an overall text rather than capturing opinions toward COVID-19–specific aspects chosen by domain experts and exploit lexicon built in general domains, overlooking that a word’s sentiment depends on the domain or context where it is used [20].

**Table 1:** Related work on topic modeling and sentiment analysis on COVID-19–related data.

Authors	Source	Posters	Time	Location	Language	Sentiment
Liu et al [10]	News articles	News reporters	January 1 to February 20, 2020	Not specified	Chinese	No
Dong et al [11]	Research papers	Researchers	Unknown to March 20, 2020	Not specified	English	No
Stokes et al [12]	Reddit posts	Public	March 3-31, 2020	Not specified	English	No
Sha et al [13]	Tweets	State governors, presidential cabinet members, and the president	January 1 to April 7, 2020	US	English	No
Hosseini et al [14]	Tweets	Public	March 13 to April 19, 2020	Iran	Persian and Farsi	No
Sharma et al [15]	Tweets	Public	March 1-30, 2020	Not specified	English	Yes
Odlum et al [16]	Tweets	Public (African Americans)	January 21 to May 3, 2020	Not specified	English	Yes
Wang et al [17]	Tweets	Public	March 5 to April 2, 2020	California and New York, US	English	Yes
Abd-Alrazaq et al [18]	Tweets	Public	February 2 to March 15, 2020	Not specified	English	Yes
Ordun et al [19]	Tweets	Public	March 24 to April 9, 2020	Not specified	English, Spanish, Italian, French, and Portuguese	No
This study	Tweets	Public	January 21 to May 31, 2020	Canada and US	English	Yes

Our study aims to investigate Twitter users’ reactions to COVID-19 in North America, especially in Canada. We analyzed COVID-19–related tweets with topic modeling and aspect-based sentiment analysis (ABSA) using human-in-the-loop and interpret the results with public health experts. We examined the sentiment of tweets about COVID-19–related aspects such as social distancing and masks by using ABSA based on domain-specific aspect and opinion terms. The key advantage of our study is that public health experts are actively involved in the computational process with the specific goal of informing public health interventions. Our results were interpreted by these public health experts, and we used a human-in-the-loop ABSA approach to obtain domain specific aspect and opinion terms. To the best of our knowledge, we are the first to directly identify sentiment of COVID-19–specific aspects.

## Methods

### Data and Data Processing

We used a public Twitter data set about the COVID-19 pandemic, collected by Chen et al [21] using numerous COVID-19–related keywords such as “coronavirus,” “COVID-19,” and “pandemic.” The data collection started on January 28, 2020 (tweets from January 21, 2020), and is still ongoing, which has published over 123 million tweets as of May 11, 2020. This data set includes retweets, quoted tweets, and replies to tweets.

For our study, we collected tweets until the end of May 2020, the end of the first wave in Canada, since we aim to investigate people’s reactions and concerns in the early days of COVID-19. We selected tweets whose location is Canada or the United States.

Among the 372,711 tweets in total (Canada: n=30,235, US: n=342,476), we only included tweets written in English using tweet metadata and the spacy-langdetect toolkit [22]. This process resulted in 319,524 tweets in total, 25,595 for Canada, and 293,929 for the United States. To remove tweet-specific keywords and URLs, we used the tweet-preprocessor toolkit [23]. We did not remove hashtags and mentions because they can be informative for our study. We lowercased and tokenized using the Spacy toolkit [24]. Since the methods we used in this paper are all unsupervised, we did not split the data for training and test. Our scripts are available on GitHub [25].

### Topic Modeling 

We first discovered topics in COVID-19–related tweets using a widely used topic modeling approach, latent Dirichlet allocation (LDA) [26]. We chose to use LDA because it is simple and popular. We also tried another popular topic modeling method, nonnegative matrix factorization, but LDA results were more distinct in categories according to public health experts’ assessment. As we have seen the potential of topic modeling in this study, we will also consider more sophisticated topic modeling algorithms such as pachinko allocation or hierarchical LDA, which allow modeling relations between topics. To assess changes in topics of discussion over time, we compared timelines of topic distributions and timing of public health intervention implementations for COVID-19.

To discover topics and track the topic change over time, we constructed topic models on our Twitter data using LDA implementation in the scikit-learn package [27]. We chose a model with 20 topics among 5, 10, 20, and 50 because 20 topics showed diverse and less redundant topics when manually examined.

The topics generated by LDA were interpreted and labeled by two public health experts. Both experts have extensive experiences in public health with doctoral training in the field. In the initial phase of the study before choosing a final model, they discussed the results to build consensus. After the final output was obtained, the junior expert interpreted and labeled it first, and the senior expert reviewed.

To analyze the dynamics of public health relevant topics, we investigated the change in the prevalence of the topics over time. More specifically, we performed a basic analysis based on an examination of the estimates of *θ*, a document-to-topic distribution, produced by the model. We first divided tweets into weekly buckets using Coordinated Universal Time–12 time stamps (eg, January 21-26, January 27 to February 2, and February 3-9, 2020). We then computed a mean *θ* vector for tweets in each bucket as done by Griffiths and Steyvers [28].

### ABSA

To capture sentiment revealed in tweets toward important aspects of COVID-19, we used ABSA. In our study, aspects can include public health interventions or issues associated with COVID-19, such as “social-distancing,” “reopening,” and “masks.” We investigated people’s opinion (positive and negative) toward these aspects.

We used ABSApp, a weakly-supervised ABSA system [29]. We chose ABSApp because it does not require labeled data for training and allows manually editing domain-specific aspect and opinion lexicons produced by the method. This feature is particularly beneficial for us because, in collaboration with domain experts, we could select and add aspects public health agencies are interested in.

The two public health experts who labeled topics from topic modeling also edited the terms so that aspect terms are related to important public health interventions or issues they are interested in and that opinion terms are words that describe sentiment of those public health terms. Similarly to the topic interpretation process, the junior expert edited the terms first, and the senior expert reviewed.

## Results

### Context

**Figure 1:**
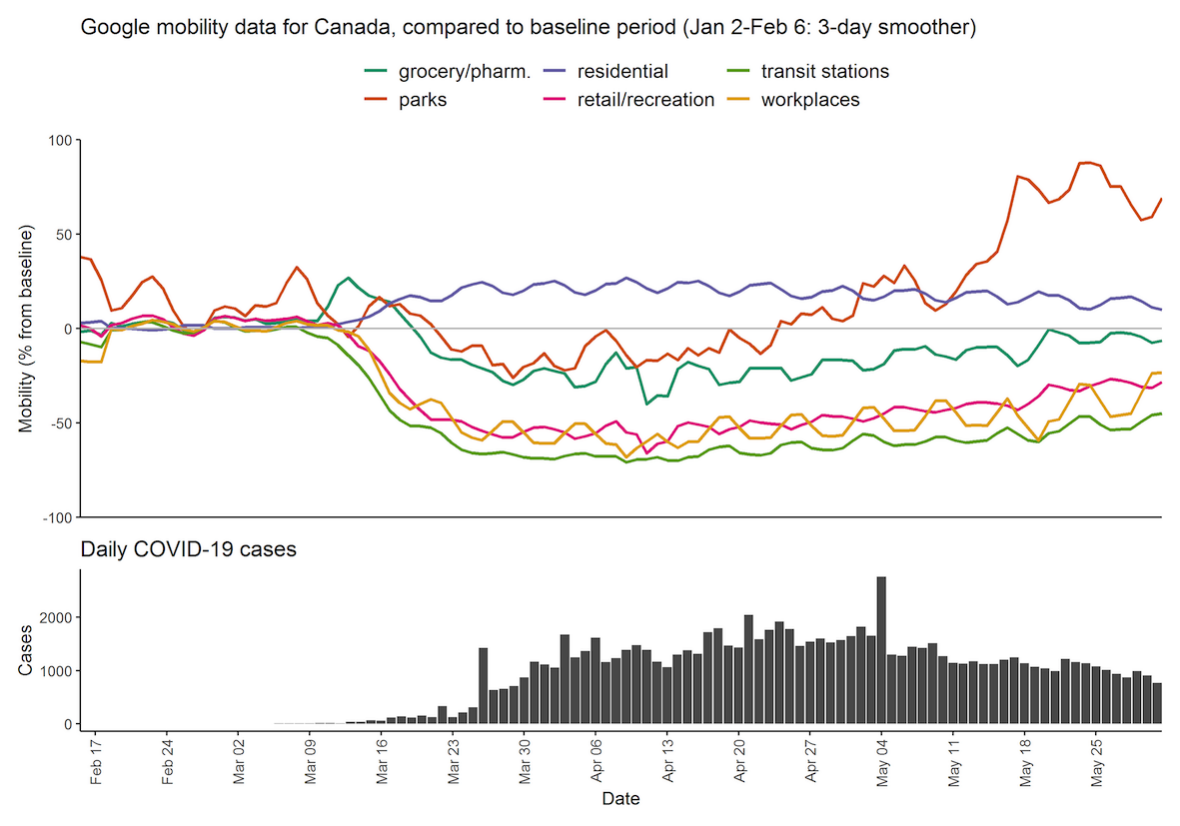
Mobility and case count for Canada from February 15 to May 31, 2020. Google mobility data is only available since February 15. pharm.: pharmacy.

**Figure 2:**
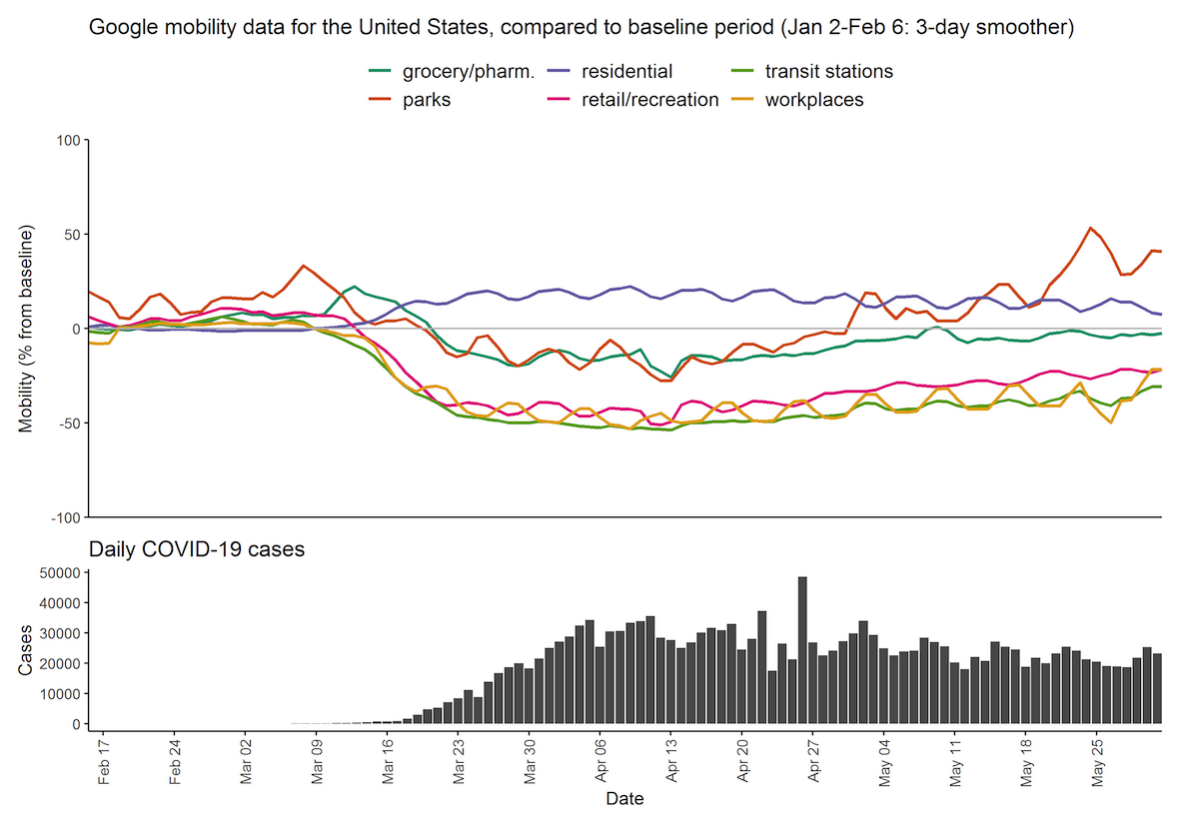
Mobility and case counts for the United States from February 15 to May 31, 2020. Google mobility data is only available since February 15. pharm.: pharmacy.

Figures 1 and 2 provide context for our results, presenting mobility and case counts for Canada and the United States. These data show that, as the daily COVID-19 cases increased, activities such as recreational or work-related mobility drastically decreased in the middle of March 2020. Around the middle of March, public health measures were put in place as well. The dates of public health orders differed by provinces or states as well as the specifics of the orders, but in Canada, on March 11, 2020, health officials in British Columbia underlined the importance of social distancing and urged people to stay home as much as possible. On March 16, 2020, an order prohibiting gatherings of 50 people or more was placed. In Ontario, a state of emergency was declared on March 17, 2020, and social distancing measures commenced. On the same day, the federal government announced closure of the Canada-US border to all nonessential traffic. In the United States, mandatory stay-at-home orders were issued, beginning in California on March 19 followed by many other states afterwards.

### Topic Modeling

The discovered topics were highly related to public health promotions and interventions such as physical distancing, border restrictions, handwashing, staying home, and face coverings, as shown in Textbox 1. Other topics included US President Donald Trump, initial outbreaks in Wuhan, economic concerns, and negative reactions. The entire set of topics is listed in Multimedia Appendix 1.

**Top 5 prevalent topics in Canada and the United States.**

**Canada**

1. Age and COVID-19 transmission, as well as time
2. Initial outbreak in Wuhan
3. US President Trump’s statement
4. Thank you notes related to the pandemic mixed with discussion of cruise ship outbreaks
5. Air travel and regional border restrictions and outbreaks

**United States**

1. Age and COVID-19 transmission, as well as time
2. US President Trump’s statement
3. Early debate on whether COVID-19 is like the flu.
4. Initial outbreak in Wuhan
5. The need to stay home and the impact of COVID-19 on essential workers and family

The most prevalent topics in Canada and the United States showed some differences, as can be seen in Textbox 1. In both countries, age and COVID-19 transmission was the most prevalent topic. The discussion around the initial outbreak in Wuhan and US President Trump’s statement was also active in both countries. However, the topic about air travel and regional border restrictions was highly ranked only in Canada, whereas the topic was not even listed in the top 10 in the United States. Similarly, the topics about COVID-19 being like the flu and staying home were highly ranked in the US tweets, but ranked lower than other topics in the Canadian tweets.

**Figure 3:**
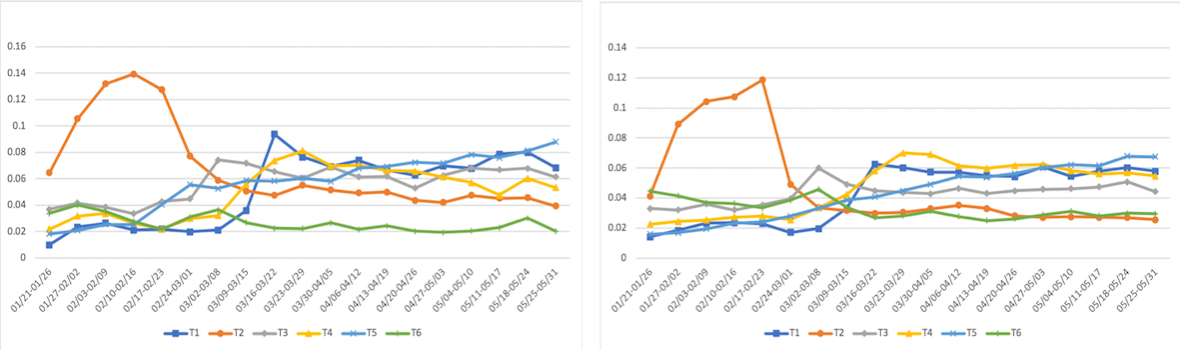
Changes of six public health–relevant topics over time. T1: social and physical distancing; T2: air travel and regional border restrictions and outbreaks; T3: handwashing and preventive measures; T4: the need to stay home and impact of COVID-19 on essential workers and family; T5: number of tests and cases; T6: masks and face coverings.

Based on the mean *θ* vector for each bucket, we drew graphs of public health–relevant topics over time as shown in Figure 3. First, we observed that the patterns in the US tweets and Canadian tweets were similar. Although there were slight differences, the overall increase and decrease patterns were almost identical. For example, the topic about air travel and regional border restrictions (T2) shows a peak in February and drastically decreases.

Second, we could see that the topic trend is highly related to public health interventions. For example, the topic about social distancing (T1) started to increase in early March 2020 after social distancing measures were enacted. Handwashing (T3) also started to be emphasized then. The topic about the need to stay home (T4) started to increase around the end of March. In Canada, the Federal Quarantine Order was issued on March 24, and in the United States, many states issued stay-at-home orders around that time as well. Discussion about the number of tests and cases (T5) gradually increased. Interestingly, the topic about masks and face coverings (T6) slightly decreased from March; this is possibly because public health institutes in both countries announced their position about masks around that time.

### Aspect-Based Sentiment Analysis

**Figure 4:**
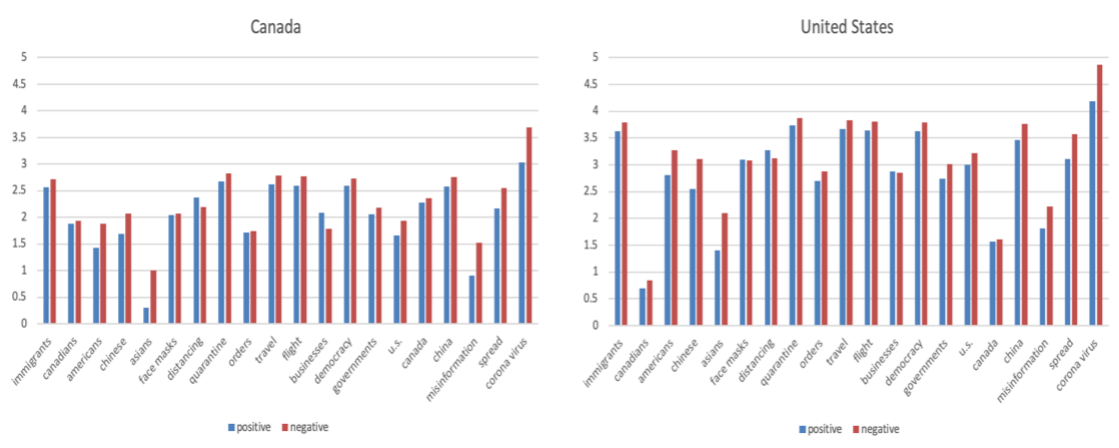
Aspect-based sentiment analysis results. x-axis: selected aspects; y-axis: number of positive occurrences and number of negative occurrences in log scale.

**Figure 5:**
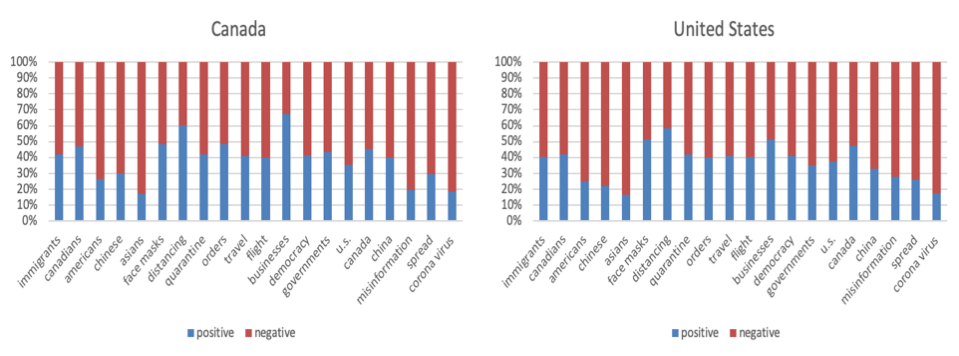
Aspect-based sentiment analysis results for selected aspects. y-axis: the ratio between number of positive occurrences and number of negative occurrences.

After training the tweet data using ABSApp, we obtained 806 aspect terms and 211 opinion terms. Manually editing the lexicons resulted in 545 aspect terms (eg, “vaccines,” “economy,” and “masks”) and 60 domain-specific opinion terms such as “infectious” (negative) and “professional” (positive). These manually edited terms were then used for the inference of sentiments for 20 key aspects selected by public health experts. The results are shown in Figures 4 and 5. Overall, the sentiments between Canada and the United States showed similar patterns. We observed that the sentiments about COVID-19 itself was dominantly negative. With this, the Twitter users’ reactions to misinformation appeared to be more negative than positive, suggesting the frustration about the situation and misinformation. The mixed sentiments about masks might reflect the conflicting messaging around using masks. The negative sentiments toward Asians may imply that anti-Asian sentiments escalated due to COVID-19.

To further investigate the possible stigma for Asians, we observed words that frequently co-occurred with the aspect words Chinese and Asians. The top-ranked words in negative tweets included “virus,” “racist,” “racism,” “fucking,” “attacks,” “ass,” “assaults,” “blame,” and “hate,” and the top-ranked words in positive tweets included “fucking,” “racism,” “respectful,” “kind,” “street,” “disgusting,” and “crying.” We list sample tweets that show positive and negative sentiments in Textbox 2.

**Sample tweets showing positive or negative sentiments toward Asians.**

**Positive**

- “You should not be afraid of Asians but you should be absolutely terrified of the PEOPLE THAT DONT COVER THEIR MOUTHS/NOSES DURING A COUGH AND/OR SNEEZE.”
- “French Asians hit back at racism with 'I'm not a virus”
- “Y’all realize that the coronavirus ain’t exclusive to Chinese people right?? mfs look for any excuse to be racist bruh”

**Negative**

- “Oriental Asians always starting some fuckin outbreak...”
- “Yea I’m holding my breath round all Asians till this coronavirus shit clear up call it wat u think it is.”
- “No Asians allowed in my shop after the outbreak.”

**Figure 6:**
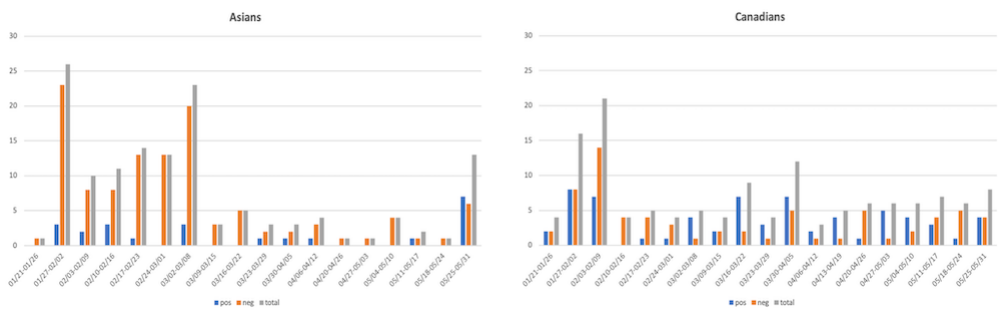
Sentiment changes over time for Asians and Canadians. Y-axis shows the number of positive occurrences, number of negative occurrences, and number of total occurrences. neg: negative; pos: positive.

Figure 6 displays sentiment changes over time toward Asians and Canadians. Although sentiments about Canadians were overall more positive except in February 2020 and after the middle of May, sentiments about Asians were mostly negative. Especially in the beginning of March when COVID-19 started to be serious in North America, we can see a spike in the number of negative tweets about Asians, and then it drastically reduces after that, which might suggest that there were some campaigns or awareness about anti-Asian racism.

## Discussion

### Principal Findings

In this study, using topic modeling and ABSA on Twitter data from North America, we identified various topics related to physical distancing, travel and boarder restrictions, handwashing and preventive measures, face masks, stay-at-home orders, and the number of cases and testing. Travel and border restrictions were major discussion points in February 2020, which were taken over by other topics such as physical distancing later in time. ABSA analysis identified various negative themes related to the overall outbreak, anti-Asian racism and misinformation, and positive occurrences related to physical distancing. These data demonstrate Twitter users’ focus on discussing and reacting to public health interventions during the first phase of the pandemic.

This kind of information could help public health agencies to understand public concerns as well as what public health messages are resonating in our populations who use Twitter. For example, public health agencies in North America have focused their messaging around encouraging hand hygiene, limiting physical contact when sick, and staying home to prevent infection. We can see this messaging echoing in the topics around handwashing, staying home, mask wearing, and social or physical distancing.

For public health decision makers, it would be beneficial to have the pipeline where a computational model keeps running on social media data as a stream, and the results are reviewed by public health experts. This will then be reflected in public health education communication or messages to address misinformation related to the topics.

Risk communication and knowledge translation in practice is a combination of proactive and reactive messaging [30,31]. Topic modeling can inform social media priorities and form a key rapid response function for public health communicators. Two key functions can be imagined: identifying new topics for public health communicators and assessing uptake of public health messages. The first function of developing new topics would follow the traditional knowledge translation cycle (in other words, identifying a knowledge gap, assessing barriers to use, developing products, disseminating, and iterating). When topic modeling identifies discourse either on aspects public health is not messaging on or misunderstandings of current health messages, this can start the knowledge translation cycle. In particular, this will help identify new and emerging areas for misinformation messaging. The second function is assessment of public health message uptake. We can explore whether key public health messages are showing up in social media discourse. If not, we can explore what is showing up instead.

Our findings that tweets reflect public health interventions are aligned with other studies. Abd-Alrazaq et al [18] performed topic modeling on tweets before mid-March 2020, and their results focused more on the virus itself (eg, its origin, impact on people, and the economy) but did not show conversations about public health interventions. However, in the studies using tweets from March and April, topics related to social distancing policies such as school closure, stay-at-home orders, and work from home commonly emerged in tweets posted by US governors and presidential cabinet executives [13]; Reddit posts [12]; tweets in English, Spanish, Italian, French, and Portuguese [19]; tweets in California and New York [17]; and tweets in Iran [14].

Depending on tweets used for analysis, other studies report some interesting topics different from topics drawn from tweets in Canada. For example, topics related to government and political issues were observed in the studies on tweets by US governors [13] and on tweets in Iran [14], whereas our analysis only showed Trump’s statement as a topic rather than overall political issues regarding COVID-19.

Our ABSA provides sentiments toward specific aspects by considering sentence structures, while most prior works performing sentiment analysis use algorithms to decide a sentiment of an entire text. For this reason, these studies are generally not suitable for identifying a sentiment of a given aspect. For instance, Wang et al [17] computed the average sentiment scores of tweets by each day and each hour rather than obtaining sentiments for aspects. Yin et al [32] related sentiment for each tweet to the topic the tweet belonged to and then investigated the overall sentiment of each topic. Therefore, it is not straightforward to compare our ABSA results with other sentiment analysis results.

However, our ABSA results, especially related to racism and discrimination against Asians, were also observed in other research using different study methods. Zhu [33] qualitatively analyzed 1366 tweets to examine swears around “Chinese virus” in multiple languages. Topic modeling on English tweets in March and April 2020 [34] showed a topic related to racism with top-ranked words such as “Chinese” and “pig.” A survey in the United States also showed prejudicial attitudes among Americans toward Chinese Americans [35]. These findings show that ABSA has the potential to track stigma and other negative consequences related to COVID-19. Our communities of Asian ethnicity have experienced unprecedented stigma and discrimination due to COVID-19. Chinese Canadians and other East Asians are experiencing hatred expressed as assaults, verbal threats, and feeling unsafe in the society. As our analysis suggests, if we monitor the change in discrimination over time using social media as a stream in real time, we could develop counteracting messages and measures in specific geographic areas whenever there is a spike in such incidents.

Our study had the following limitations. We used only a small set of Twitter data because tweets with the location information were limited compared to the whole data set. This has affected other studies using social media data in a similar fashion. Moreover, it should be noted that the geo-tagged tweets data set comprises statements from a nonuniform subsample of the population. According to Gore et al [36], only 15% of online adults regularly use Twitter, and those aged 18-29 years and minorities tend to be more highly represented on Twitter than in the general population.

In our data set, we looked at location at the country level (ie, Canada or the United States). However, Gore et al [36] showed that there could be significant geographic bias at the city level in the sentiment expressed in tweets over the same time period. Therefore, there may be a risk that specific geographic areas at the city level might be overrepresented for a given country in our study.

Another possible bias comes from not knowing who tweeted from the locations. Padilla et al [37] showed that the sentiment of tweets could be biased based on if people are local or visiting an area at the time of their tweets. Our data set could be biased in this regard. However, given travel restrictions and use of country instead of city, this bias may not be an issue for this analysis.

In general, whenever our proposed pipeline would be deployed in practice, all these biases should be carefully considered and addressed.

In addition, although ABSA allows capturing more nuanced sentiments toward specific aspects, it also has the limitation that current state-of-the-art sentiment analysis techniques have: it cannot properly handle figurative languages such as sarcasm. However, since our proposed approach can process substantial amounts of twitter data, it should be able to deal with the noise generated by these complex pragmatic phenomena.

### Conclusion

In this paper, we present the exploratory results of topic modeling and ABSA on COVID-19–related tweets in North America, especially in Canada. We compared topic modeling and ABSA results of Canada and the United States, and showed public health intervention–related topic changes over time. Our analyses demonstrated that Twitter conversations about COVID-19 are highly aligned with public health interventions. In our study, public health experts were actively involved in the computational process as well as interpretation of the results. The human-in-the-loop ABSA allowed manually editing aspect and opinion lexicons, and as a result, our analysis showed sentients toward the aspects public health experts were interested in by leveraging the domain-specific lexicons. Our results suggest that monitoring Twitter user’s reactions about COVID-19–related aspects can be beneficial for public health policy makers.

## Appendix

Multimedia Appendix 1 Latent Dirichlet allocation–generated topics and their interpretations.

## Abbreviations

ABSA: aspect-based sentiment analysis
LDA: latent Dirichlet allocation
