# Supplementary material for: Tracking COVID-19 Discourse on Twitter in North America: Infodemiology Study Using Topic Modeling and Aspect-Based Sentiment Analysis
Source: J Med Internet Res. 2021 Feb 10;23(2):e25431. doi: 10.2196/25431 (PMC7879725; doi:10.2196/25431)
Supplement: Multimedia Appendix 1 [file jmir_v23i2e25431_app1.docx]

Table 1. LDA generated topics and their interpretations.

| # | Representative words | Interpretation |
| --- | --- | --- |
| T1 | social, distancing, outside, park, walk, quarantinelife, stayhome | Social and physical distancing, including spending time outside during quarantine. |
| T2 | corona, beer, stupid, die, cure, flu, drink, cold | Early debate on whether coronavirus is like the flu and around corona beer sales. |
| T3 | china, travel, canada, russia, flights, trade, border | Air travel and regional border restrictions/outbreaks. |
| T4 | hands, wash, health, public, use, need, safety | Hand washing and what people can do to prevent COVID-19. |
| T5 | home, stay, safe, work, sick, family, essential | The need to stay home and the impact of COVID-19 on essential workers and family. |
| T6 | positive, testing, tested, cases, patients, hospital, data | This topic focuses on data, particularly number of tests and cases. |
| T7 | masks, wear, face, hand, sanitizer, gloves, n95 | Things we can do to prevent COVID-19, e.g., masks and face coverings. |
| T8 | trump, china, americans, hoax, cdc, democrats, pandemic | US President Trump’s statement of whether COVID-19 is a hoax and his discussion of China. |
| T9 | students, pandemic, nyc, petition, climate, college, university | A mix of discussion around school closures, the climate and the outbreak in New York City. |
| T10 | trump, house, white, president, press, vote, conference | USA politics including the white house press conferences and the US election. |
| T11 | test, cdc, facts, vaccine, fake, lab, control | Lab testing for COVID-19 and vaccination discussions, as well as discussing ‘fake’ tests available. |
| T12 | china, cases, death, wuhan, outbreak, spread, rate | Initial outbreak in Wuhan and its associated case and death statistics. |
| T13 | time, old, years, day, feel, life, long | A mix of discussion around age and COVID-19 transmission, as well as time. |
| T14 | cases, deaths, york, million, state, total, cuomo | The statistics around deaths, particularly cases and deaths in New York City. |
| T15 | thanks, help, support, cruise, community, team, proud | Thank you notes related to the pandemic mixed with discussion of cruise ship outbreaks. |
| T16 | health, need, care, public, emergency, fighting, stigma, curve, job | The need for health care related to addressing the COVID-19 pandemic in the USA. |
| T17 | money, pay, paper, toilet, buying, water, price | General economic concerns including pay and bulk buying. |
| T18 | stayhome, lockdown, order, quarantine, florida, beach, california | Quarantine and lockdown orders, particularly in Florida beaches and California. |
| T19 | shit, fucking, ass, wow, damn, dumb, hell | Negative reactions to COVID-19 and emotional usage of swearing. |
| T20 | break, trip, spring, school, classes, summer, quarnantinelife | COVID-19 school closures and spring break. |
